# Sex differences in psychiatric comorbidities of attention-deficit/hyperactivity disorder among children, adolescents, and adults: A nationwide population-based cohort study

**DOI:** 10.1371/journal.pone.0315587

**Published:** 2025-01-03

**Authors:** Pei-Hsin Kao, Chung-Han Ho, Charles Lung-Cheng Huang

**Affiliations:** 1 Department of Psychiatry, Chi Mei Medical Center, Tainan, Taiwan; 2 Department of Medical Research, Chi Mei Medical Center, Tainan, Taiwan; 3 Department of Information Management, Southern Taiwan University of Science and Technology, Tainan, Taiwan; 4 Department of Medicinal and Applied Chemistry, Kaohsiung Medical University, Kaohsiung, Taiwan; Jawaharlal Institute of Postgraduate Medical Education and Research, INDIA

## Abstract

This cross-sectional, nationwide, population-based study aimed to elucidate sex differences in psychiatric comorbidities of Attention-deficit/hyperactivity disorder (ADHD) across children, adolescents, and adults. We analyzed data from Taiwan’s comprehensive healthcare database, including 112,225 individuals diagnosed with ADHD, categorized by age (0–12, 13–18, ≥18 years) and sex. Psychiatric comorbidities were assessed using ICD-9-CM codes, focusing on age and sex-specific prevalence. Among the cohort, 83.50% were children (0–12 years) presenting primarily with learning disabilities and tics, while adolescents (13–18 years; 11.88%) had higher instances of oppositional defiant and conduct disorders. In adults (≥18 years; 4.62%), the prevalence of anxiety, depression, bipolar disorder, and substance misuse was notably higher. Males under 18 predominantly had ADHD, whereas females exhibited increased vulnerability to emotional disorders. In adulthood, males showed greater susceptibility to most psychiatric comorbidities, except sleep disorders. The study highlights the evolving nature of ADHD-related psychiatric comorbidities across different life stages, with distinct sex-based patterns. The transition from childhood to adulthood sees an increased prevalence of various psychiatric conditions, particularly impacting adult males. These findings underscore the need for age- and sex-specific therapeutic approaches in ADHD management. The cultural context of the study necessitates further research in diverse populations for broader applicability of the findings.

## Introduction

Attention-deficit/hyperactivity disorder (ADHD) is a prevalent neurobehavioral disorder characterized by inattention, hyperactivity, and impulsivity, leading to significant morbidity [1]. These symptoms impact various aspects of an individual’s life, including academic performance [2, 3], social relationships [4], self-esteem, and accident risk. Additionally, ADHD can cause challenges in occupational settings and overall daily functioning, contributing to a reduced quality of life. The etiology of ADHD is multifactorial, involving genetic, environmental, and biological factors. It affects individuals across all sexes and often continues from childhood into adulthood [5, 6]. Approximately 60% of children diagnosed with ADHD continue to experience symptoms into adulthood [7], with about 5% of children and 2.5% of adults affected globally [8].

ADHD is categorized into three subtypes based on predominant symptoms: predominantly inattentive presentation (difficulties in sustaining attention, frequent careless mistakes, easily distracted), predominantly hyperactive-impulsive presentation (fidgeting, inability to stay seated, excessive talking, interrupting others), and combined presentation (significant levels of both inattention and hyperactivity-impulsivity) [9]. In this study, "sex" refers to biological attributes, while "gender" refers to societal roles, with our analysis focusing on sex differences due to the biological aspects of ADHD.

The pervasive presence of psychiatric comorbidities complicates ADHD management, challenging therapeutic strategies, reducing diagnostic clarity, and increasing healthcare costs [10, 11]. Early studies focused on pediatric comorbidities, such as mood and anxiety disorders, oppositional defiant disorder, conduct disorder, learning disabilities, and substance use disorder [12], Recent studies, however, have highlighted the persistence of ADHD and its associated comorbidities across the lifespan [13, 14].

The presentation of ADHD symptoms varies among individuals and across different life stages, regardless of the presence of co-occurring disorders. The challenges posed by ADHD and its comorbidities change as individuals progress through life stages. In childhood, the focus is on foundational learning; during adolescence, complex peer dynamics become more prominent; and in adulthood, individuals with ADHD face socioeconomic and familial responsibilities [15, 16]. Notably, there are nuanced sex-based variations in the prevalence and prognosis of ADHD [15]. For instance, women with ADHD are more likely to be diagnosed with comorbid anxiety, depression, bipolar disorder, and personality disorders, while men with ADHD have a higher prevalence of schizophrenia and substance use [15].

In light of these complexities, our study of a representative cohort with ADHD from Taiwan aimed to illuminate the intricacies of this landscape. We had three core objectives: firstly, to elucidate the psychiatric comorbidities of individuals with ADHD across age groups and to observe the variations in these comorbidities between age groups; secondly, to probe into sex-centric disparities in each age group; and finally, to evaluate the overarching influence of sex on ADHD comorbidity profiles. We aim to equip clinicians with nuanced insights that foster more individualized therapeutic strategies.

## Materials and methods

### Data source

Taiwan’s National Health Insurance Research Database (NHIRD) contains healthcare data of approximately 99% of Taiwan’s population, covered by the National Health Insurance program initiated in 1995. Managed by the National Health Research Institutes, the NHIRD consolidates data from various healthcare services. The datasets are linked via encrypted personal identification numbers to ensure confidentiality. For research purposes, the data is further de-identified to ensure individual identities cannot be traced while still allowing a comprehensive overview of an individual’s health information.

### Study design and individuals

This study is a cross-sectional analysis of individuals diagnosed with ADHD between January 2000 and December 2011, identified from the NHIRD. In Taiwan, ADHD is predominantly diagnosed by psychiatrists based on clinical interviews, scale evaluations, and sometimes neuropsychological tests such as continuous performance tests. We used the International Classification of Diseases, Ninth Revision, Clinical Modification (ICD-9-CM) code 314 for ADHD. To ensure a confirmed diagnosis of ADHD, we required at least one hospitalization or three outpatient visits for the condition. This criterion was chosen to ensure robust confirmation of diagnosis. While this may result in a sample that includes more severe cases of ADHD compared to other countries where fewer visits are required, it allows for a higher diagnostic accuracy. Outpatient visits were defined as visits to a physician for diagnosis and evaluation. Participants were grouped by age (0–12, 13–18, and ≥18 years) to reflect developmental stages and differing healthcare needs. Significant differences in comorbidities among these age cohorts were assessed and are discussed in the results section. The age groups were further subdivided by sex to examine sex differences in comorbidities. This study utilized data from a 12-year period, collected from various healthcare providers. Each participant was assigned a unique identifier to prevent multiple representations. Data were de-identified for privacy. The study focused on diagnosing ADHD and comorbid psychiatric conditions, with detailed medical histories recorded for each participant. The NHIRD provides a comprehensive and representative cohort because it includes nearly the entire population of Taiwan, covering diverse demographic groups. This broad coverage ensures that our findings are generalizable to the broader Taiwanese population, reflecting a wide range of ADHD cases across different age groups and sexes. We accessed the NHIRD data for research purposes between February 13, 2014, and March 15, 2015. Despite the specific timeframe for data access, the NHIRD dataset’s comprehensive nature and depth provide valuable insights into ADHD and its comorbidities over the studied period.

### Comorbidities

We examined 11 comorbidities, each identified by one or more ICD-9-CM codes: learning disabilities (315), tics (307.2), oppositional defiant disorder (313.81), conduct disorder (312), anxiety disorder (300.0x, 300.2x, 300.3), depressive disorder (296.2x, 296.3x, 300.4, and 311), bipolar disorder (296.0x, 296.1x, 296.4x, 296.5x, 296.6x, 296.7x, 296.80, 296.81, 296.89), adjustment disorder (309.x), sleep disorders (307.4 and 780.5), alcohol use disorder (303), and substance use disorder (303). We required at least one hospitalization or three outpatient visits for the condition for a confirmed diagnosis.

### Ethical statements

Our study complied with Chi Mei Medical Center’s ethical standards and secured appropriate approvals. The IRB/ethics committee waived the requirement for consent because we utilized deidentified secondary data from the NHIRD. The NHIRD data is initially linked via encrypted personal identification numbers to ensure confidentiality, and then further deidentified for research purposes to protect individual privacy. This waiver has no impact on the rights and welfare of the participants. In addition, we confirm that all methods in our study were performed in accordance with the relevant guidelines and regulations, including adherence to the principles of the Declaration of Helsinki.

### Statistical analysis

Sex and comorbidities are expressed as numbers and percentages. Differences in distributions between age categories and sexes were evaluated using Pearson’s chi-square test. Logistic regression was used to compare sex-comorbidity associations between age groups and provide odds ratios (ORs) with 95% confidence intervals (CIs). This method allows us to control for potential confounding variables (e.g., age, sex) and estimate the strength of association between ADHD and its comorbidities across different subgroups. By providing ORs, we can quantify the likelihood of comorbidities in different sex and age groups. Separate analyses were conducted for sex and age to explore their individual effects on comorbidities. Additionally, interactions between age and sex were tested to understand their combined effect on comorbidity patterns. Given the large number of statistical tests conducted, we applied the Bonferroni correction to account for multiple testing. Bar charts were used to visually compare psychiatric disorder categories between the three age groups stratified by sex, with error bars representing the standard error of the mean (SEM) to provide a clear understanding of the data variability. Only positive error bars were displayed to avoid visual clutter. A *p*-value < 0.05 was considered statistically significant. Statistical analyses were performed using SAS 9.4 for Windows (SAS Institute, Cary, NC, USA).

## Results

### Comorbidities by age group

**Fig 1:**
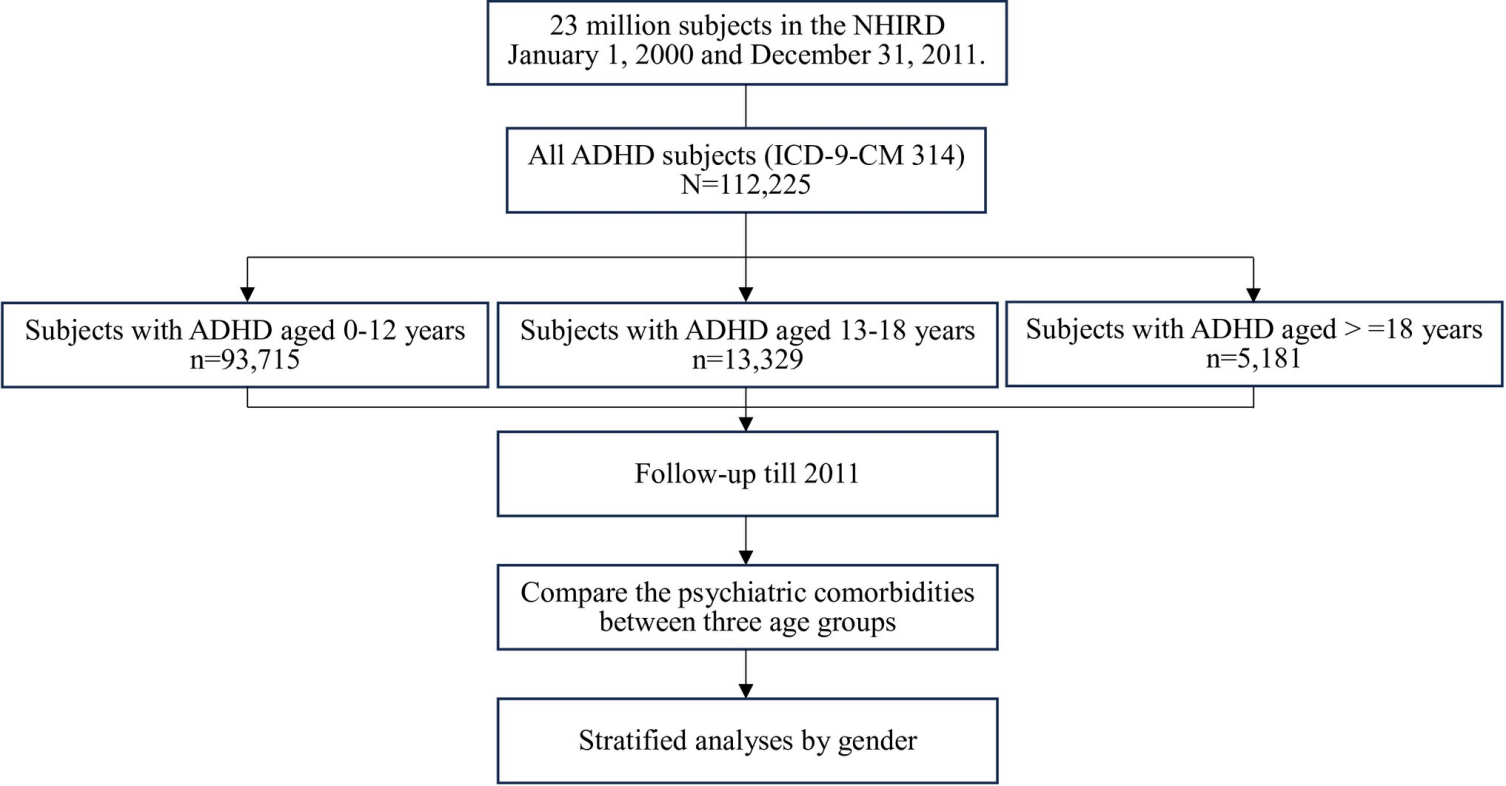
Study flowchart of psychiatric comorbidities in ADHD by age. This figure illustrates the cross-sectional analysis of comorbidities in individuals with ADHD across different age groups at a single point in time. NHIRD, National Health Research Database; ADHD, Attention Deficit Hyperactivity Disorder.

We evaluated data for 112,225 individuals newly diagnosed with ADHD over 12 years (Fig 1).

They were categorized into three age groups: 0–12 years (83.50%), 13–18 years (11.88%), and ≥18 years (4.62%) (Table 1).

**Table 1:** Characteristics and psychiatric comorbid conditions among subjects with ADHD in three age groups.

Age, years				Overall N = 112,225 (%)	0–12 years n = 93,715 (%)	13–18 years n = 13,329 (%)	> = 18 years n = 5,181 (%)	p-value
**Gender**		**Male**		87,562 (78.02)	74,708 (79.72)	10,782 (80.89)	2,072 (39.99)	< .0001
		**Female**		24,663 (21.98)	19,007 (20.28)	2,547 (19.11)	3,109 (60.01)	
**Comorbidity**								
	**Learning disability**		**No**	65,120 (58.03)	48,892 (52.17)	11,187 (83.93)	5,041 (97.30)	< .0001
			**Yes**	47,105 (41.97)	44,823 (47.83)	2,142 (16.07)	140 (2.70)	
	**Tics**		**No**	105,218 (93.76)	87,577 (93.45)	12,547 (94.13)	5,094 (98.32)	< .0001
			**Yes**	7,007 (6.24)	6,138 (6.55)	782 (5.87)	87 (1.68)	
	**Oppositional defiant disorder**		**No**	90,117 (80.30)	74,900 (79.92)	10,192 (76.46)	5,025 (96.99)	< .0001
			**Yes**	22,108 (19.70)	18,815 (20.08)	3,137 (23.54)	156 (3.01)	
	**Conduct disorder**		**No**	105,660 (94.15)	88,768 (94.72)	11,951 (89.66)	4,941 (95.37)	< .0001
			**Yes**	6,565 (5.85)	4,947 (5.28)	1,378 (10.34)	240 (4.63)	
	**Anxiety disorder**		**No**	83,210 (74.15)	71,614 (76.42)	9,111 (68.35)	2,485 (47.96)	< .0001
			**Yes**	29,015 (25.85)	22,101 (23.58)	4,218 (31.65)	2,696 (52.04)	
	**Depressive disorder**		**No**	103,544 (92.26)	89,834 (95.86)	10,667 (80.03)	3,043 (58.73)	< .0001
			**Yes**	8,681 (7.74)	3,881 (4.14)	2,662 (19.97)	2,138 (41.27)	
	**Bipolar disorder**		**No**	109,978 (98.00)	92,736 (98.96)	12,753 (95.68)	4,489 (86.64)	< .0001
			**Yes**	2,247 (2.00)	979 (1.04)	576 (4.32)	692 (13.36)	
	**Adjustment disorder**		**No**	104,479 (93.10)	88,344 (94.27)	11,725 (87.97)	4,410 (85.12)	< .0001
			**Yes**	7,746 (6.90)	5,371 (5.73)	1,604 (12.03)	771 (14.88)	
	**Sleep disorder**		**No**	97,087 (86.51)	83,955 (89.59)	10,823 (81.20)	2,309 (44.57)	< .0001
			**Yes**	15,138 (13.49)	9,760 (10.41)	2,506 (18.80)	2,872 (55.43)	
	**Alcohol use disorder**		**No**	112,063 (99.86)	93,690 (99.97)	13,293 (99.73)	5,080 (98.05)	< .0001
			**Yes**	162 (0.14)	25 (0.03)	36 (0.27)	101 (1.95)	
	**Drug use disorder**		**No**	112,018 (99.82)	93,641 (99.92)	13,292 (99.72)	5,085 (98.15)	< .0001
			**Yes**	207 (0.18)	74 (0.08)	37 (0.28)	96 (1.85)	

*p-value is from the Pearson’s chi-squared (χ2) tests

The overall mean age of participants was 9.19±7.08 years. The sex breakdown of participants was 78.02% males and 21.98% females. ADHD was significantly more prevalent among males aged <18 years compared to females (p < 0.001), but this trend reversed in adults, with more females being diagnosed with ADHD compared to males. The prevalence of learning disabilities and tics were notably high in the youngest age group (0–12 years) of individuals with ADHD and declined with age. Among children aged 0–12 years, 47.83% of males and 45.12% of females had learning disabilities, which decreased to 2.70% in adult males and 3.10% in adult females. The prevalence of tics decreased from 6.55% in children to 1.68% in adults, with males consistently showing higher rates across all age groups. The prevalence of oppositional defiant disorder and conduct disorder was higher among adolescents (13–18 years). In contrast, the prevalence of anxiety, depressive, bipolar, adjustment, sleep disorders, and substance use disorder increased with age, being most prevalent in adults (≥18 years).

### Sex differences in comorbidities

The likelihood of comorbidities in individuals with ADHD was related to sex (Table 2).

**Table 2:** Odds ratio of comorbidities between male and female subjects with ADHD.

Comorbidity			Males n = 87,562 (%)	Females n = 24,663 (%)	p-value	Odds ratio (95% C.I.) Males vs. Females
	**Learning disability**	**No**	50,581 (57.77)	14,539 (58.95)	0.0009	1.00 (ref.)
		**Yes**	36,981 (42.23)	10,124 (41.05)		1.05 (1.02–1.08)
	**Tics**	**No**	81,264 (92.81)	23,954 (97.13)	< .0001	1.00 (ref.)
		**Yes**	6,298 (7.19)	709 (2.87)		2.62 (2.42–2.83)
	**Oppositional defiant disorder**	**No**	70,053 (80.00)	20,064 (81.35)	< .0001	1.00 (ref.)
		**Yes**	17,509 (20.00)	4,599 (18.65)		1.09 (1.05–1.13)
	**Conduct disorder**	**No**	82,171 (93.84)	23,489 (95.24)	< .0001	1.00 (ref.)
		**Yes**	5,391 (6.16)	1,174 (4.76)		1.31 (1.23–1.40)
	**Anxiety disorder**	**No**	65,176 (74.43)	18,034 (73.12)	< .0001	1.00 (ref.)
		**Yes**	22,386 (25.57)	6,629 (26.88)		0.93 (0.91–0.96)
	**Depressive disorder**	**No**	81,648 (93.25)	21,896 (88.78)	< .0001	1.00 (ref.)
		**Yes**	5,914 (6.75)	2,767 (11.22)		0.57 (0.55–0.60)
	**Bipolar disorder**	**No**	85,920 (98.12)	24,058 (97.55)	< .0001	1.00 (ref.)
		**Yes**	1,642 (1.88)	605 (2.45)		0.76 (0.69–0.84)
	**Adjustment disorder**	**No**	81,751 (93.36)	22,728 (92.15)	< .0001	1.00 (ref.)
		**Yes**	5,811 (6.64)	1,935 (7.85)		0.83 (0.79–0.88)
	**Sleep disorder**	**No**	76,940 (87.87)	20,147 (81.69)	< .0001	1.00 (ref.)
		**Yes**	10,622 (12.13)	4,516 (18.31)		0.62 (0.59–0.64)
	**Alcohol use disorder**	**No**	87,426 (99.84)	24,637 (99.89)	0.0683	1.00 (ref.)
		**Yes**	136 (0.16)	26 (0.11)		1.47 (0.97–2.24)
	**Drug use disorder**	**No**	87,402 (99.82)	24,616 (99.81)	0.7999	1.00 (ref.)
		**Yes**	160 (0.18)	47 (0.19)		0.96 (0.69–1.33)

*p-value is from the Pearson’s chi-squared (χ2) tests. Bonferroni correction was applied to adjust for multiple comparisons.

Males showed higher risks of learning disabilities, tics, oppositional defiant disorder, and conduct disorder but lower risks of comorbid anxiety, depressive, bipolar, adjustment, and sleep disorders. The differences in comorbid alcohol use disorder and substance use disorder risks between sexes were negligible.

### Effects of the interaction between age and sex on comorbidities

The sex breakdown of these individuals with ADHD, along with the number of participants with co-occurring pathologies, is detailed in Table 3.

**Table 3:** Odds ratio of comorbidity between male and female subjects with ADHD in three age groups.

			0–12 years n = 93,715 (%)			13–18 years n = 13,329 (%)			> = 18 years n = 5,181 (%)		
**Comorbidity**			Males n = 74,708	Females n = 19,007	OR (95% C.I.) M vs. F	Males n = 10,782	Females n = 2,547	OR (95% C.I.) M vs. F	Males n = 2,072	Females n = 3,109	OR (95% C.I.) M vs. F
	**Learning disability**	**No**	39,560 (52.95)	9,332 (49.10)	1.00 (ref.)	9,043 (83.87)	2,144 (84.18)	1.00 (ref.)	1,978 (95.46)	3,063 (98.52)	1.00 (ref.)
		**Yes**	35,148 (47.05)	9,675 (50.90)	0.86 (0.83–0.88)	1,739 (16.13)	403 (15.82)	1.02 (0.91–1.15)	94 (4.54)	46 (1.48)	3.16 (2.21–4.52)
	**Tics**	**No**	69,184 (92.61)	18,393 (96.77)	1.00 (ref.)	10,079 (93.48)	2,468 (96.90)	1.00 (ref.)	2,001 (96.57)	3,093 (99.49)	1.00 (ref.)
		**Yes**	5,524 (7.39)	614 (3.23)	2.39 (2.20–2.60)	703 (6.52)	79 (3.10)	2.18 (1.72–2.76)	71 (3.43)	16 (0.51)	6.86 (3.98–11.83)
	**Oppositional defiant disorder**	**No**	59,813 (80.06)	15,087 (79.38)	1.00 (ref.)	8,270 (76.70)	1,922 (75.46)	1.00 (ref.)	1,970 (95.08)	3,055 (98.26)	1.00 (ref.)
		**Yes**	14,895 (19.94)	3,920 (20.62)	0.96 (0.92–1.00)	2,512 (23.30)	625 (24.54)	0.93 (0.84–1.03)	102 (4.92)	54 (1.74)	2.93 (2.10–4.09)
	**Conduct disorder**	**No**	70,625 (94.53)	18,143 (95.45)	1.00 (ref.)	9,653 (89.53)	2,298 (90.22)	1.00 (ref.)	1,893 (91.36)	3,048 (98.04)	1.00 (ref.)
		**Yes**	4,083 (5.47)	864 (4.55)	1.21 (1.13–1.31)	1,129 (10.47)	249 (9.78)	1.08 (0.93–1.25)	179 (8.64)	61 (1.96)	4.72 (3.51–6.35)
	**Anxiety disorder**	**No**	56,782 (76.01)	14,832 (78.03)	1.00 (ref.)	7,461 (69.20)	1,650 (64.78)	1.00 (ref.)	933 (45.03)	1,552 (49.92)	1.00 (ref.)
		**Yes**	17,926 (23.99)	4,175 (21.97)	1.12 (1.08–1.17)	3,321 (30.80)	897 (35.22)	0.82 (0.75–0.90)	1,139 (54.97)	1,557 (50.08)	1.22 (1.09–1.36)
	**Depressive disorder**	**No**	71,733 (96.02)	1,8101 (95.23)	1.00 (ref.)	8,869 (82.26)	1,798 (70.59)	1.00 (ref.)	1,046 (50.48)	1,997 (64.23)	1.00 (ref.)
		**Yes**	2,975 (3.98)	906 (4.77)	0.83 (0.77–0.89)	1,913 (17.74)	749 (29.41)	0.52 (0.47–0.57)	1,026 (49.52)	1,112 (35.77)	1.76 (1.57–1.97)
	**Bipolar disorder**	**No**	73,905 (98.93)	1,8831 (99.07)	1.00 (ref.)	10,354 (96.03)	2,399 (94.19)	1.00 (ref.)	1,661 (80.16)	2,828 (90.96)	1.00 (ref.)
		**Yes**	803 (1.07)	176 (0.93)	1.16 (0.99–1.37)	428 (3.97)	148 (5.81)	0.67 (0.55–0.81)	411 (19.84)	281 (9.04)	2.49 (2.11–2.93)
	**Adjustment disorder**	**No**	70,528 (94.40)	17,816 (93.73)	1.00 (ref.)	9,561 (88.68)	2,164 (84.96)	1.00 (ref.)	1,662 (80.21)	2,748 (88.39)	1.00 (ref.)
		**Yes**	4,180 (5.60)	1,191 (6.27)	0.89 (0.83–0.95)	1,221 (11.32)	383 (15.04)	0.72 (0.64–0.82)	410 (19.79)	361 (11.61)	1.88 (1.61–2.19)
	**Sleep disorder**	**No**	67,055 (89.76)	16,900 (88.91)	1.00 (ref.)	8,910 (82.64)	1,913 (75.11)	1.00 (ref.)	975 (47.06)	1,334 (42.91)	1.00 (ref.)
		**Yes**	7,653 (10.24)	2,107 (11.09)	0.92 (0.87–0.96)	1,872 (17.36)	634 (24.89)	0.63 (0.57–0.70)	1,097 (52.94)	1,775 (57.09)	0.85 (0.76–0.95)
	**Alcohol use disorder**	**No**	74,685 (99.97)	19,005 (99.99)	1.00 (ref.)	10,751(99.71)	2,542 (99.80)	1.00 (ref.)	1,990 (96.04)	3,090 (99.36)	1.00 (ref.)
		**Yes**	23 (0.03)	2 (0.01)	2.93 (0.69–12.41)	31 (0.29)	5 (0.20)	1.47 (0.57–3.77)	82 (3.96)	19 (0.61)	6.70 (4.06–11.07)
	**Drug use disorder**	**No**	7,4651 (99.92)	18,990 (99.91)	1.00 (ref.)	1,0750 (99.70)	2,542 (99.80)	1.00 (ref.)	2,001 (96.57)	3,084 (99.20)	1.00 (ref.)
		**Yes**	57 (0.08)	17 (0.09)	0.85 (0.50–1.47)	32 (0.30)	5 (0.20)	1.51 (0.59–3.89)	71 (3.43)	25 (0.80)	4.38 (2.77–6.93)

*p-value is from the Pearson’s chi-squared (χ2) tests. Bonferroni correction was applied to adjust for multiple comparisons.

**Fig 2:**
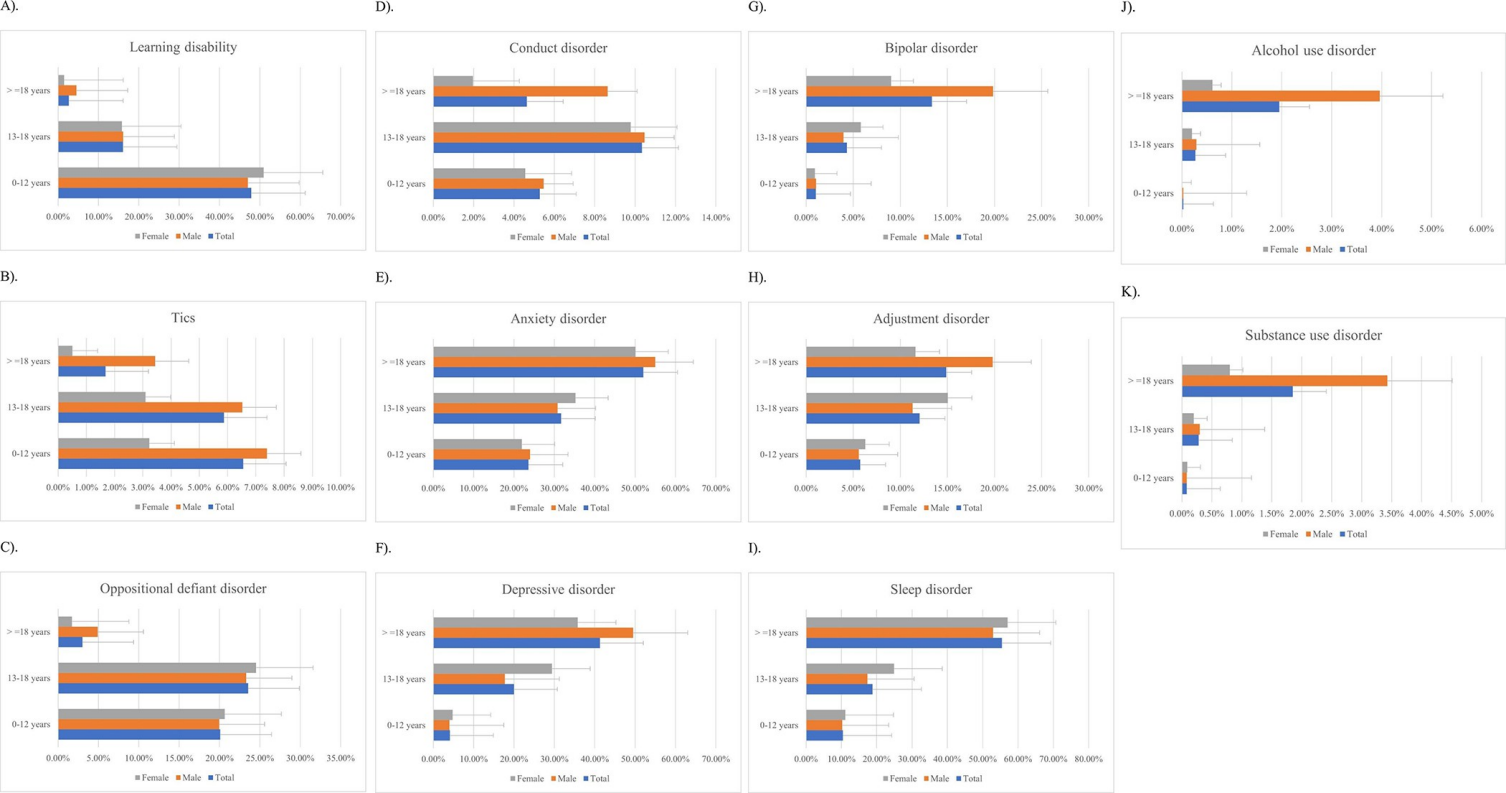
Comorbidity rate of various psychiatric disorders among total, male, and female subjects with ADHD. Error bars represent the standard error of the mean (SEM) and only the positive error bars are shown. A). Learning disability B). Tics C). Oppositional defiant disorder D). Conduct disorder E). Anxiety disorder F). Depressive disorder G). Bipolar disorder H). Adjustment disorder I). Sleep disorder J). Alcohol use disorder K). Substance use disorder.

Among children (0–12 years) with ADHD, males had a lower risk of learning disabilities than females, but this trend reversed among adults. Tics were more prevalent in males, regardless of age. The risk of comorbid oppositional defiant disorder was higher for adult males. While males generally showed an increased tendency for comorbid conduct disorder compared to females, this difference decreased in adolescence (13–18 years). The prevalence of emotional disorders (including depressive, and bipolar disorders) also differed between sexes. Males aged <12 years or >18 years were more likely to be affected by comorbid anxiety than females in these age groups, but among adolescents, females showed a slightly increased risk of anxiety. The trend in comorbid depression mirrored this pattern, with adult males showing heightened susceptibility. The risk of severe disorders such as comorbid bipolar disorder was lower for adolescent males but higher for adult males. Males aged ≥18 years with ADHD were distinctly prone to comorbid alcohol use disorder and substance use disorder. Fig 2 show the marked impact of psychiatric comorbidities, except sleep disorders, on adult males with ADHD.

## Discussion

Our comprehensive, nationwide study in Taiwan investigated ADHD-associated comorbidities across various age groups. Consistent with previous studies [17], we found that ADHD was more prevalent among males, yet we uncovered nuanced patterns of comorbidity.

### Age and sex dynamics

Our data show a reversal in the male-to-female ADHD ratio from childhood to adulthood, possibly due to under-recognition of ADHD in young females and reduced help-seeking behavior among adult males [17]. Referral biases contribute to under-diagnosis in younger females, as their symptoms and clinical presentations often differ from those of males. Sex differences in symptom expression, neurocognition, and comorbidity also likely shape the healthcare-seeking patterns in ADHD [17]. Studies from Japan and Korea observed a narrowing of the sex difference in ADHD prevalence from childhood to adulthood [18, 19]. Unlike our findings, these studies did not show a reversal in the male-to-female ratio but did report a reduced sex gap. These differences may arise from cultural variations in help-seeking behaviors, which could impact regional diagnostic rates. However, the studies only explored age-related trends in ADHD prevalence and did not assess sex-specific changes in comorbidities across different ages, from childhood into adulthood in their cohorts.

### Age-related comorbidities

The prevalence of learning disabilities and tics decreased with age, while oppositional defiant disorder and conduct disorder peaked during adolescence. Conversely, anxiety, depression, bipolar disorder, adjustment disorders, sleep disturbances, and substance use disorder increased with age, being most prevalent in adults. Notably, our study found ADHD to be significantly more prevalent among males under the age of 18 (p < 0.001) based on Pearson’s chi-square test. However, in adulthood (≥18 years), more females were diagnosed with ADHD compared to males, contrary to the established 1.6:1 male-to-female ratio commonly reported in the literature [20]. This discrepancy may be due to the severity criteria used in our study, which might have influenced diagnosis patterns.

### Sex-based comorbidity patterns

Sex-based analyses revealed that males with ADHD were more likely to suffer from learning disabilities, tics, and behavioral disorders, whereas females exhibited higher rates of emotional and sleep disorders. Particularly, adult males faced the most significant impacts of comorbidities, except for sleep disorders. This study is unique in examining the interaction between age and sex on ADHD comorbidities, providing a novel perspective on these dynamics throughout an individual’s life.

### Comorbid with learning disabilities and tics

Our study highlights the age-related pattern of ADHD coexisting with learning disabilities and tics. In the 0–12-year age group, 47.83% of children diagnosed with ADHD also had learning disabilities, decreasing to 2.70% in adults, while the prevalence of tics decreased from 6.55% in children to 1.68% in adults. These findings are consistent with those of Burgić Radmanović and Burgić (2020) [21] and Seo et al. (2022) [18], which observed similar trends in the U.S. and Korea, showing that comorbidity typically declines with age.

Shared genetics, symptomatology, and neurological anomalies underpin the overlap between ADHD, learning disabilities, and tics [22, 23]. For instance, deficits in fronto-striatal circuits, which improve with age, might be responsible [24]. Additionally, genes such as TBC1D7 further complicate the relationship between ADHD and tics [25, 26]. As individuals age, the co-occurrence of these conditions becomes less pronounced, possibly due to adaptive coping strategies, neurodevelopment, and targeted interventions.

Consistent with Burgić Radmanović and Burgić (2020) [21], our findings show that males with ADHD have higher learning disabilities and Tourette’s disorder rates across age groups. Boys with ADHD and tics often experience heightened anxiety and reduced social adeptness [11]. ADHD negatively impacts academic achievements, leading to underperformance and increased risk of early school dropout [2, 3]. Optimal interventions for ADHD can alleviate symptoms and manage tic manifestations effectively.

### Comorbid with oppositional defiant disorder and conduct disorder

Oppositional defiant disorder frequently coexists with ADHD in early childhood, with comorbidity rates reaching 50%–60% in children and adolescents with ADHD [27–29]. A Norwegian study found oppositional defiant disorder in 31% of children with ADHD, surpassing the 10% prevalence of conduct disorder [30]. Our results align with these findings, showing oppositional defiant disorder prevalence at 20.08% in the 0–12-year age group, peaking at 23.54% during adolescents, and then decreasing to 3.01% in adults. Conduct disorder rates were 5.28% in children, 10.34% in adolescents, and 4.63% in adults. Studies, including one from Korea [18], consistently highlight a post-adolescence decline in both oppositional defiant disorder and conduct disorder.

The connections between ADHD and oppositional defiant disorder/conduct disorder are complex, involving shared genes like the dopamine receptor D4 (DRD4) gene, and environmental conditions such as family dysfunction [31]. Genes like solute carrier family 6 member 4 (SLC6A4) and fronto-striatal abnormalities also contribute [32, 33]. These genetic elements, merged with environmental and social risk factors, explain high comorbidity rates and their evolution with age. Hormonal and neural transitions during adolescence might exacerbate ADHD symptoms, while neural maturation and effective interventions may reduce these co-occurrences in adults [34–36].

Our analysis highlights distinct sex variations. While oppositional defiant disorder prevalence in individuals with ADHD aged <18 years did not differ by sex, adult males showed higher oppositional defiant disorder rates. Conduct disorder was consistently more prevalent among males with ADHD across age groups, although some studies reported differences in these comorbidities [30, 37]. The simultaneous presence of ADHD and oppositional defiant disorder or conduct disorder intensifies symptoms and complicates treatment outcomes.

### Comorbid with anxiety, depressive, bipolar, adjustment, and sleep disorders

Our study explored the complex coexistence of ADHD with several psychiatric disorders, highlighting an age-dependent increase in comorbidity rates, especially in adults. Anxiety disorders in subjects with ADHD increased from 23.58% in children to 52.04% in adults, consistent with previous studies [14, 18]. Depressive disorders (41.27% in adults) and bipolar disorder (13.36% in adults) also showed similar trends [14]. The clinical relevance of adjustment disorders was evident, with a 14.88% rate in adults. Sleep disturbances affected 55.43% of adults with ADHD, aligning with reported rates of 25%–55% [38].

A network of genetic and environmental factors contributes to the interplay between ADHD and psychiatric conditions like anxiety and depression. These overlaps may arise from psychosocial stressors, impacts of preceding disorders, and complex biological underpinnings [39]. Anxiety can sometimes mask ADHD, complicating diagnoses, while depression in ADHD might stem from attenuated hedonic sensations [14]. Genetic research has highlighted the shared bases of ADHD and depression, with studies emphasizing the continuity of childhood ADHD into adult ADHD and depression [40, 41]. The coexistence of ADHD and bipolar disorder is also rooted in shared genetics and environmental factors [42, 43].

Individuals with ADHD face amplified emotional problems due to academic and social challenges. Childhood sleep disturbances, often exacerbated by familial conflicts, predict subsequent ADHD diagnoses [11]. Medication side effects sometimes cause sleep disruptions, necessitating a nuanced treatment approach balancing sleep hygiene and appropriate medication [38, 42].

Our study emphasizes the pivotal role of sex in ADHD comorbidity dynamics. Adult females with ADHD often present with anxiety, depression, and bipolar disorder, a trend supported by previous research [13, 44]. Shared genetic factors and heightened societal pressures related to inattention might intensify their vulnerability [3]. ADHD in females—marked by inattention without obvious hyperactivity—is often misdiagnosed and/or not diagnosed [45]. In contrast, adult males with ADHD manifest higher rates of comorbidities, except for sleep disturbances. Potential underdiagnosis in females and male-focused presentations might contribute to these observed sex disparities [46, 47]. Societal norms emphasizing male resilience and inherent biological differences between males and females may also be influential.

The complexity of ADHD escalates when paired with other psychiatric conditions. Children with ADHD and depression face magnified social and academic challenges [4]. Depressive episodes in ADHD are marked by heightened severity, extended duration, and increasing suicidal tendencies. The overlap between ADHD and bipolar disorder complicates the diagnostic and therapeutic landscapes. Stimulants, a cornerstone of ADHD treatment, can pose risks, such as mood destabilization, in those with comorbid bipolar disorder [48]. Additionally, the association between ADHD and sleep disturbances profoundly affects mood, behavior, and overall well-being.

### Comorbid with alcohol use disorder and substance use disorder

The relationship between ADHD and alcohol use disorder or substance use disorder varies between studies. For instance, McGough et al. reported a lifetime alcohol use disorder prevalence of 34% in adults with ADHD [44], while Taurines et al. reported 25% [11]. In contrast, our study, consistent with Seo et al. [18], identified a modest alcohol use disorder prevalence of 1.95% in adults with ADHD, with minimal sex differences. A previous Taiwanese study found substance use disorder prevalences of 21.7% and 14.6% in adults with early- and late-onset ADHD, respectively [39]. Our study indicates a substance use disorder prevalence of 1.85%, suggesting potential underdiagnosis of substance use disorder in Taiwan, likely due to societal stigmas.

Genetic, neurobiological, and environmental factors underlie the co-occurrence of ADHD and alcohol use disorder or substance use disorder. Genetic predisposition to externalizing behaviors can steer individuals with ADHD toward alcohol use disorder and substance use disorder [3]. The genetics of ADHD, alcohol use disorder, and substance use disorder show significant overlaps, particularly in neurotransmission pathways [11, 49]. Maternal alcohol consumption during pregnancy is a substantial environmental risk, potentially elevating the child’s likelihood of developing ADHD [42].

Sex significantly influences the dynamics between ADHD and alcohol use disorder or substance use disorder. While Luderer et al. [49] highlighted increased alcohol abuse in females with ADHD, our data present a contrasting narrative. The sex difference in alcohol use disorder is negligible up to 18 years of age; however, alcohol use disorder is markedly more prevalent among males in adulthood. In the case of substance use disorder, cultural factors might drive females with ADHD toward different coping strategies despite inherent risks of substance neurotoxicity.

The overlap between ADHD and alcohol use disorder or substance use disorder presents diagnostic challenges, as alcohol misuse can mask genuine ADHD manifestations. Achieving alcohol use disorder stability is paramount for unambiguous ADHD assessment [50]. The convergence of ADHD and substance use disorder complicates diagnosis, with affected individuals facing greater challenges, including elevated suicide risk and treatment complexities [51, 52].

Addressing these interconnected challenges is vital for effective ADHD treatment. Clinicians must prioritize stabilizing substance use disorder to pinpoint genuine ADHD symptoms [50]. Selecting the appropriate treatment for ADHD, weighing stimulant against non-stimulant options, is crucial for those comorbid with ADHD and substance use disorder. A comprehensive approach integrating psychosocial strategies is essential for optimizing treatment outcomes for this group [53].

### Strengths and limitations

Our study possesses several significant strengths that enhanced the current understanding of ADHD and its associated comorbidities. Utilizing a nationwide, population-based dataset enabled a broad and comprehensive analysis of ADHD and its comorbidities. By probing the trajectories of psychiatric comorbidities of males and females across different age groups, we provided novel insights into the temporal complexities of ADHD comorbidities. This can pave the way for specialized therapeutic strategies and informed policy measures. A notable strength of our study is its focus on sex-specific complexities of ADHD comorbidities, a domain often overlooked in previous studies.

However, our study had inherent limitations. Firstly, the criterion for ADHD diagnosis in our sample required at least three outpatient visits or one hospitalization, potentially resulting in a sample that includes more severe cases of ADHD. In many countries, fewer visits are required for an ADHD diagnosis, and hospitalization for ADHD is very rare. Therefore, our sample may not fully represent the general ADHD population and may over-represent more severe cases. This should be considered when interpreting the results, as the observed comorbidity patterns may be influenced by the severity of the cases included. Secondly, although we determined correlations between ADHD and numerous conditions, we did not examine potential confounding variables such as socioeconomic dynamics, lifestyle choices, and genetic predispositions. This introduces complexity to our conclusions. Additionally, the number of participants with multiple co-occurring pathologies (e.g., ADHD, anxiety disorder, and depressive disorder) was not analyzed, which could provide further insights into the complexity of ADHD and its comorbid conditions. Thirdly, our reliance solely on diagnoses recorded in a database might have introduced inaccuracies. The database did not provide explicit details on treatment adherence, which is pivotal for identifying comorbidity progression. Our study is also subject to the potential for underdiagnosis, a perennial concern in psychiatric research, as unreported instances might lead to an underestimation of prevalence. Fourthly, although we conducted separate analyses for sex and age and tested for age-sex interactions, there may still be unaccounted influences due to the complex nature of these interactions. The application of the Bonferroni correction to address multiple testing issues, while necessary, does not entirely eliminate the risk of Type I errors due to the large number of statistical tests conducted. Future studies should aim to conduct more comprehensive interaction analyses and consider alternative methods to further control for multiple comparisons. Fifthly, our study is a cross-sectional analysis, capturing a snapshot of ADHD comorbidities across different age groups at a single point in time. This design allows us to compare the prevalence of comorbidities between age groups and sexes, but it does not establish causal relationships or track changes over time, as would be possible in a longitudinal study. This constraint limits our understanding of the progression of ADHD and the interplay between its causes and outcomes. Furthermore, our study could not determine whether symptoms in the adult ADHD population began in childhood or emerged only in adulthood. This inability to distinguish between these subgroups restricts our insights into the nuanced differences in comorbidities among them. Nevertheless, ADHD is widely recognized as a neurodevelopmental disorder, present from birth. This perspective is well-documented in major diagnostic manuals [9, 54], emphasizing that ADHD symptoms often manifest early in development and can persist throughout an individual’s lifespan. These symptoms may vary in form and intensity due to factors such as symptom severity, environmental influences, individual differences, and the awareness and availability of diagnosis. Recognizing ADHD as a chronic condition underscores the importance of effective identification and treatment across all life stages. Finally, a salient point to consider is the potential cultural specificity of our findings. While our results are comprehensive within the Taiwanese milieu, they may not generalize to other cultural landscapes due to differences in ADHD diagnostic and treatment practices. Future research may benefit from comparing findings from diverse cultural settings.

## Conclusions

ADHD demonstrates a multifaceted trajectory marked by dynamic comorbidities across different life stages. From early-onset learning impairments to anxiety, depressive, and substance-related disorders in adulthood, the course of ADHD is intertwined with various mental and behavioral afflictions. Our study highlights the age-related patterns of comorbidities: learning disabilities and tics are more prevalent in younger children; oppositional defiant disorder and conduct disorder peak during adolescence; while anxiety, depressive, bipolar, adjustment, and sleep disorders, as well as substance use disorder, become more common in adulthood. Sex-specific differences also play a crucial role in the manifestation of comorbidities. Males are more prone to learning disabilities, tics, oppositional defiant disorder, and conduct disorder, whereas females exhibit higher rates of emotional and sleep disorders. Our findings indicate that adult males with ADHD face a more pronounced burden of comorbidities, necessitating sex-specific therapeutic strategies. A complex blend of biological, psychological, and environmental determinants drives the varied nature of ADHD. While the foundational symptoms of ADHD might decrease with age, the ensuing challenges, amplified by life events such as academic endeavors and vocational pursuits, persist for many. The inclusion of error bars in our figures helps to illustrate the variability and reliability of the comorbidity rates among different age and sex groups, providing a clearer picture of the data and supporting the robustness of our findings.

Our results underscore the urgent need for versatile therapeutic strategies. These approaches should recognize the evolving landscape of comorbidities, particularly given the clear ramifications for adult males within our research scope. The nuanced dynamics of personal relationships further bolster the case for a comprehensive, patient-centric therapeutic paradigm encompassing both the individual and their familial milieu. Ultimately, adept navigation of ADHD demands an in-depth grasp of its shifting landscape of comorbidities to pave the way for prompt and efficacious interventions across all life stages.
